# RNA-Seq Differentiates Tumour and Host mRNA Expression Changes Induced by Treatment of Human Tumour Xenografts with the VEGFR Tyrosine Kinase Inhibitor Cediranib

**DOI:** 10.1371/journal.pone.0066003

**Published:** 2013-06-19

**Authors:** James R. Bradford, Matthew Farren, Steve J. Powell, Sarah Runswick, Susie L. Weston, Helen Brown, Oona Delpuech, Mark Wappett, Neil R. Smith, T. Hedley Carr, Jonathan R. Dry, Neil J. Gibson, Simon T. Barry

**Affiliations:** 1 Oncology, AstraZeneca Pharmaceuticals, Alderley Park, Cheshire, United Kingdom; 2 Personalised Healthcare and Biomarkers, AstraZeneca Pharmaceuticals, Alderley Park, Cheshire, United Kingdom; 3 Oncology, AstraZeneca Pharmaceuticals, Gatehouse Park, Massachusetts, United States of America; Philipps University, Germany

## Abstract

Pre-clinical models of tumour biology often rely on propagating human tumour cells in a mouse. In order to gain insight into the alignment of these models to human disease segments or investigate the effects of different therapeutics, approaches such as PCR or array based expression profiling are often employed despite suffering from biased transcript coverage, and a requirement for specialist experimental protocols to separate tumour and host signals. Here, we describe a computational strategy to profile transcript expression in both the tumour and host compartments of pre-clinical xenograft models from the same RNA sample using RNA-Seq. Key to this strategy is a species-specific mapping approach that removes the need for manipulation of the RNA population, customised sequencing protocols, or prior knowledge of the species component ratio. The method demonstrates comparable performance to species-specific RT-qPCR and a standard microarray platform, and allowed us to quantify gene expression changes in both the tumour and host tissue following treatment with cediranib, a potent vascular endothelial growth factor receptor tyrosine kinase inhibitor, including the reduction of multiple murine transcripts associated with endothelium or vessels, and an increase in genes associated with the inflammatory response in response to cediranib. In the human compartment, we observed a robust induction of hypoxia genes and a reduction in cell cycle associated transcripts. In conclusion, the study establishes that RNA-Seq can be applied to pre-clinical models to gain deeper understanding of model characteristics and compound mechanism of action, and to identify both tumour and host biomarkers.

## Introduction

Human tumour xenografts are commonly used to model response to targeted therapeutics, and the intrinsic or acquired resistance mechanisms that can limit therapeutic benefit. Growth of these models is dependent on the interplay between the human tumour cells and murine stromal cells such as endothelial cells, leukocytes and fibroblasts recruited to generate a pro-tumour microenvironment. An ability to differentiate effects on the tumour and its surrounding tissue is critical to the development of a clinically relevant understanding of new therapeutic activity. This is of particular importance when studying agents that impact both the tumour and stroma. For example, cediranib [1], a potent vascular endothelial growth factor receptor tyrosine kinase inhibitor, reduces tumour growth by perturbing tumour-stromal interactions controlling angiogenesis.

A range of techniques have been used to gain insight into how the effects mediated by therapeutics deliver anti-tumour efficacy or to generate broad transcript profiles to assess changes following treatment. Many of these techniques such as immunohistochemistry (IHC), Enzyme-Linked Immunosorbent Assay (ELISA) or Western blotting based phenotypic or pharmacodynamic measures are limited to a small number of endpoints, such as direct target or pathway inhibition, the modulation of cell function such as proliferation or apoptosis, or changes in cell content. In contrast, hypothesis free assessments with techniques such as gene expression arrays are limited by several issues including the dynamic range of the technologies employed and species specificity [2]–[5]. These limitations compromise our ability to confidently differentiate murine from human transcripts and thereby determine the dynamic changes within each compartment of a xenograft tumour upon treatment with therapeutic agents [6].

Several statistical approaches have been devised to deconvolute expression in mixed tissue samples from microarray data. These methods are inherently sensitive to statistical assumptions, and require either prior knowledge of tissue-specific gene expression [7], a purified reference for each tissue type [8] [9], the proportions of each tissue type [10] [11] or expression data from at least one tissue type [12] [13]. An alternative is to mask human and/or mouse probe sequences defined as susceptible to cross-species hybridization prior to gene expression quantification [6] [14], demonstrating that cell type specific gene expression quantification is possible without prior cell separation although at the expense of a significant loss of data. Recently, a competitive cross-species hybridization strategy was devised that can simultaneously measure gene expression of cancer and host cells from a single xenograft tissue by hybridizing an un-manipulated RNA sample to human and mouse arrays under high stringency conditions [15]. The study reported that a combination of the Illumina BeadChip Expression array platform, specifically designed to minimize human-mouse cross-species hybridization, and specialist experimental conditions distinguished tumour from host signals with sufficient specificity and sensitivity. Nevertheless, the approach remains constrained by inherent limitations in array technology such as biased transcript coverage and limited dynamic range.

A recent study of orthology between human and mouse at the exon level revealed that only 7% of exons possess no orthologous pairings and that these are predominantly located in the UTRs [16]. However it was also shown that the degree of nucleotide sequence similarity between the remainder of orthologous exons varies between 70% and 90% with an average of 85% similarity [17] suggesting that there is sufficient RNA sequence diversity at this level to distinguish between the majority of human and mouse orthologous transcripts.

Sequencing mRNA libraries using second generation sequencing platforms (RNA-Seq) is emerging as an alternative to microarrays for genomic analysis of tumours. Sequencing offers several technical advantages over arrays including greater sensitivity and dynamic range and the avoidance of probe effects. In addition to mRNA quantification, sequence data can be used to detect gene sequence variants including splice isoforms and gene fusions, as well as somatic mutations [18]. RNA-Seq also offers unbiased quantification of all expressed genes and is not restricted to loci targeted specifically by array probesets, therefore increasing the possibility of quantifying non-protein coding genes such as lncRNA.

We have devised an RNA-Seq informatics workflow to simultaneously measure gene expression from tumour and host tissue from a human xenograft model. By applying a species-specific mapping strategy we are able to accurately distinguish RNA originating from human derived cancer cells from the RNA of murine cells. Our approach does not require prior manipulation of the RNA population, customized sequencing protocols, specific experimental conditions, or prior knowledge of the human:mouse component ratio in the RNA sample. We demonstrate that the expression values obtained from RNA-Seq in both tumour and host are comparable with species-specific RT-qPCR. Moreover we were able to demonstrate the value of this approach to detect changes in both tumour and host gene expression following treatment with cediranib.

## Results

### Species-specific RNA-Seq Workflow

**Figure 1:**
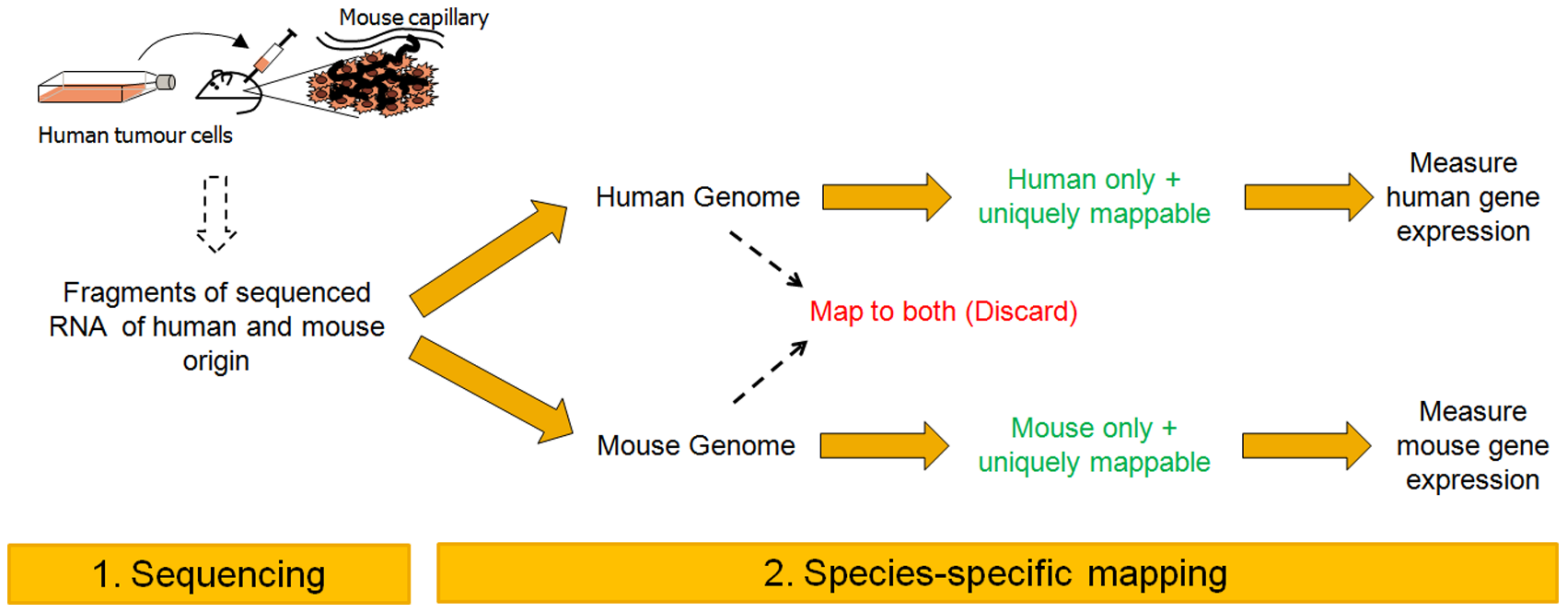
Schematic of the species-specific mapping workflow applicable to RNA-Seq data from xenografts. (1) Human tumour cells originating from the Calu-6 non-small cell lung carcinoma cell line were grown in mouse, and RNA extracted and sequenced using standard protocols. (2) Sequenced reads were then mapped to human and mouse genomes separately, and reads mapping to both species discarded. The remaining reads were used to quantify and delineate tumour (human) from host (mouse) gene expression.

RNA was isolated from four Calu-6 Non-Small Cell Lung Carcinoma (NSCLC) human xenografts: two untreated, control (vehicle-only) xenografts (referred to as Control_1 and Control_2), and two xenografts (referred to as VEGFi_1 and VEGFi_2) from mice dosed for 4 days with cediranib at 6 mg/kg once daily orally as previously described [1]. 50 bp reads obtained from the SOLiD v4 platform (Applied Biosystems) were aligned to the human (GRCh37/hg19) and mouse (NCBI37/mm9) genomes separately. To differentiate human and mouse expression it was essential to remove ∼450 K reads mapping to both human and mouse genomes resulting in loss of yields in each species of 2–2.5% for human and 17–26% for mouse. The loss of a higher proportion of mouse genes reflected the lower number of reads mapping to mouse. Reads that mapped to multiple loci on the same genome were also removed. A schematic of the species-specific mapping strategy is shown in Figure 1. Overall, ∼16–25 M and ∼1.3–2.0 M reads mapped uniquely to human and mouse respectively (Table S1), the majority mapping to known transcripts (Table S2) with most of the protein coding genes featuring at least one uniquely mappable read (Table S3). Excellent correspondence (*r^2^* = 0.92; Figure S1; Table S4) was achieved between the proportion of reads mapping to mouse and the experimentally estimated [19] (see Materials and Methods) mouse RNA composition in the sample which ranged from ∼8–17%, suggesting that the method could serve as an accurate alternative for measuring the tumour-host content of a xenograft sample and that the majority of reads are mapped to the correct species.

### Comparison of RNA-Seq with RT-qPCR

In order to validate the species-specific gene expression levels quantified by RNA-Seq, human and mouse specific assays detecting 170 human and 174 mouse genes (listed in Table S5) believed to be associated with the interaction between tumour cells and their microenvironment [19], and therefore expected to show detectable expression in these samples, were selected for RT-qPCR TaqMan® Profiling on the Fluidigm BioMark System using the same two control and treated samples analysed by RNA-Seq. The Fluidigm array was designed to incorporate probes specifically targeting human or mouse genes [19] and therefore provides a means to further assess whether reads are mapping to the correct species. Reproducibility between control biological replicates observed on both RNA-Seq (Figure S2) and RT-qPCR (Figure S3) platforms was high. Details of the assay probe identifiers and -ΔCT values across both control and treated samples are given in Table S5.

**Figure 2:**
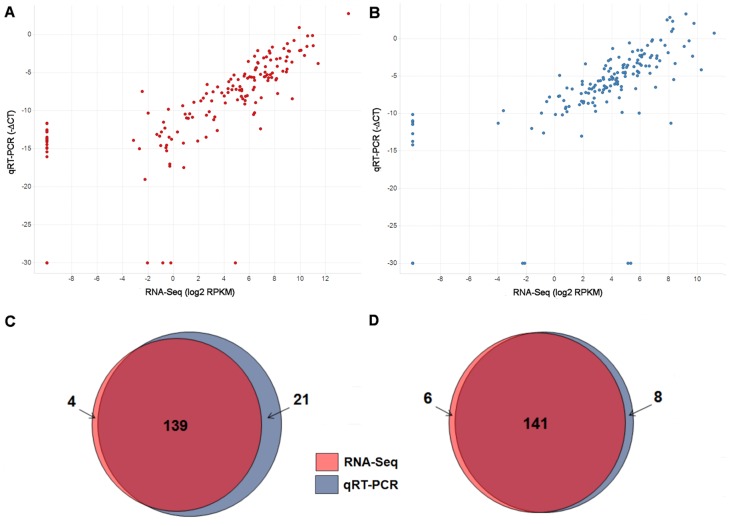
RNA-Seq gene quantification and detection closely corresponds to values obtained by species-specific RT-qPCR. Comparison between RNA-Seq and RT-qPCR across 170 human and 174 mouse genes from a Calu-6 human xenograft sample (Control_2). Correlation in gene expression in (A) human (*r^2^* = 0.72) and (B) mouse (*r^2^* = 0.60). *r^2^* values are calculated only from genes detected on both platforms. Overlap in number of (C) human and (D) mouse genes detected in both RNA-Seq and RT-qPCR, and genes detected on one platform only. The transcript mapped to by the highest number of reads was chosen to represent the expression of its parent gene. Any gene containing at least one mappable read was classed as detected in RNA-Seq. The genes included in this comparison are listed in Table S5. Details of gene expression and detection correspondence across both Control_1 and Control_2 samples are given in Table S8.

Using the Control_2 replicate as an example, significant correspondence was observed in gene expression quantification across human (*r^2^* = 0.72; Figure 2A; Table S6A). Despite the low mouse component in the RNA sample (15%), good correspondence was also achieved in mouse gene expression (*r^2^* = 0.60; Figure 2B; Table S6B). Similar levels of correlation (Table S6) were observed across the Control_1 sample (human: *r^2^* = 0.69; mouse: *r^2^* = 0.59) and two replicates treated with cediranib VEGFi_1 (human: *r^2^* = 0.69; mouse: *r^2^* = 0.56) and VEGFi_2 (human: *r^2^* = 0.62; mouse: *r^2^* = 0.56). Correspondence in gene detection was also high across both species (Table S6). Genes containing at least one mapped read were classed as detected in RNA-Seq since this threshold provided high levels of correspondence in both mouse and human (Figure S4). In Control_2, 95% (144/152) of human genes detected in RNA-Seq (with at least one mappable read) were also detected in RT-qPCR (Figure 2C). Conversely, 93% (144/155) of human genes detected in RT-qPCR were also detected in RNA-Seq. A similar pattern of correspondence was also observed in the mouse Control_2 sample in which 97% (139/143) of mouse genes detected in RNA-Seq were also detected in RT-qPCR, and 87% (139/160) detected in RT-qPCR were also detected in RNA-Seq (Figure 2D).

Of the 174 mouse genes, 13 genes were classed as differentially expressed (|log_2_ fold change|>1, *p*-value <0.05) in response to cediranib by RNA-Seq using the two control and two treated samples. In order to validate these, we performed RT-qPCR on an additional four treated and six control samples. ΔCT values for all six treated and eight control samples are given in Table S7. Table 1 indicates that 8/13 genes classed as differentially expressed by RNA-Seq were validated by RT-qPCR (|log_2_ fold change| >0.50, *p*-value<0.05). Of these, there was strong agreement (RT-qPCR |log_2_ fold change| >1.00, *p*<0.05) between platforms for four of these genes (*Flt1*, *Kdr*, *Dll4*, *Il1b*). These results suggest that RNA-Seq shows rich potential as a robust platform to measure mouse gene expression changes from xenograft samples even at low coverage and with low numbers of replicates.

**Table 1:** RT-qPCR validation of mouse gene expression changes in response to cediranib identified by RNA-Seq.

	RNA-Seq		RT-qPCR		
Gene	log_2_ FC	*p*-value	log_2_ FC	*p*-value	Agreement^1^
Vegfa	2.49	6.06E-05	0.95	4.28E-02	**
Mmp9	2.42	4.60E-04	0.36	6.64E-01	*
Flt1	−1.65	8.54E-04	−1.11	1.68E-06	***
Gpr116	−1.30	2.85E-03	−0.61	1.02E-04	**
Serpine2	−1.06	7.61E-03	0.30	7.59E-01	NA
Tie1	−1.42	7.99E-03	−0.70	1.03E-02	**
Kdr	−1.21	8.08E-03	−1.25	7.98E-06	***
Tek	−13.73	1.66E-02	−0.46	9.44E-02	**
Eng	−1.08	2.58E-02	−0.32	4.70E-01	*
Dll4	−2.84	3.40E-02	−1.67	1.27E-02	***
Il1b	1.23	3.69E-02	1.29	3.53E-02	***
Serpineb5	3.46	3.73E-02	−0.20	8.94E-01	NA
Pecam1	−1.02	4.13E-02	−0.20	5.02E-01	*

1 ***Strong agreement (RT-qPCR |log2FC|>1 and p-value<0.05),

** agreement (RT-qPCR |log2FC|>0.50 and p-value<0.05),

* some agreement (fold change in same direction), NA no agreement.

### Comparison of RNA-Seq with Human and Mouse Gene Expression Microarrays

The same two control RNA samples analysed by RNA-Seq were hybridised to Affymetrix Human U133 Plus 2.0 and Affymetrix Mouse 430 2.0 gene arrays. The majority of probes on these arrays have been designed to target the 3′ un-translated region (3′UTR) of a gene, which is typically divergent between even highly conserved ortho- and parologues [16]. This maximizes the probability of delineating expression of genes from different species making it the most appropriate standard array technology to perform species-restricted expression measurements.

**Figure 3:**
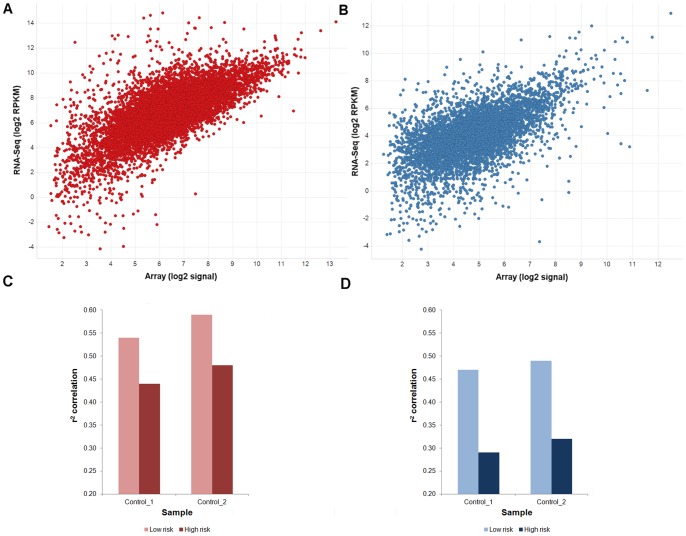
RNA-Seq versus microarray gene expression correspondence. Comparison between RNA-Seq and microarray platforms across 8621 human and 5467 mouse genes from a Calu-6 human xenograft sample (Control_2) and detected on both platforms. Correlation in gene expression in (A) human (*r^2^* = 0.50) and (B) mouse (*r^2^* = 0.35). *r* ^2^ values are calculated only from genes detected on both platforms. The transcript mapped by the highest number of reads was chosen to represent the expression of its parent gene. Any gene containing at least one mappable read was classed as detected in RNA-Seq. A gene was called “present” on the array if the signals of all probesets assigned unambiguously to that gene were separable from the general background with a *p*-value<0.01. Correlation increases between (C) human and (D) mouse gene expression values across both samples when probes on the array at high risk of cross-species hybridization are removed. Only genes detected on both platforms were considered. A mean of ∼8400 human and ∼5300 mouse genes were used in the comparison of which ∼6800 and ∼4400 genes were deemed at high risk respectively. Genes at high risk were defined as those corresponding to a probe with up to three mismatches to the transcriptome of the alternative species.

Overall, good correspondence was achieved between RNA-Seq and microarray quantified human gene expression values (*r^2^* = 0.46–0.50; Figure 3A; Table S8A). Lower correspondence was observed between mouse genes (*r^2^* = 0.32–0.35; Figure 3B; Table S8B) possibly due to the low mouse content of xenograft samples either increasing the susceptibility of the mouse array to cross-species contamination from human transcripts or reducing the ability of both platforms to quantify host expression accurately. To test the contribution of the former and estimate the impact of cross-species hybridization we identified probes at high risk of cross-species signal, defined as those with up to three mismatches to the transcriptome of the alternative species [14]. High risk human gene expression correspondence was consistently lower (mean Δ*r^2^* = −0.11; Figure 3C, Table S8A) than low risk correspondence. This decrease was more marked on the mouse array (mean Δ*r^2^* = −0.18; Figure 3D, Table S8B) suggesting that, whilst cross-species hybridization is an issue on both the human and mouse arrays, more of an effect is observed on arrays designed for the smaller species component, possibly due to greater competition from the larger component for high risk probes.

### RNA-Seq Differentiates Two Different Xenograft Models

To assess further the ability of RNA-Seq to distinguish tumour from host signals, we performed a second RNA-Seq analysis on samples from two untreated LoVo colorectal adenocarcinoma human xenografts. Mapping statistics similar to those from the Calu6 model were achieved and these are summarized in Table S9. We compared relative human and mouse gene expression levels between the Calu6 (lung) and LoVo (colon) models, hypothesizing that the majority of the expression changes would be observed between tumour rather than host genes. 4219 tumour genes were differentially expressed (log_2_ fold change magnitude >1.5, *p*<0.001) between Calu6 and LoVo compared to only 140 host genes. Genes over-expressed in LoVo were significantly enriched for associations with colorectal cancer (IPA *p* = 5.74×10^−14^) corresponding to the LoVo tumour type.

### *In-silico* Functional Enrichment Analysis of the Tumour-host Specific Response to Cediranib

We next investigated whether differentially expressed genes identified from the RNA-Seq approach reflect known biology associated with the response to the VEGF-signaling inhibitor cediranib. Cediranib is a small molecule inhibitor of VEGF-R1, 2, and 3 with additional activity versus c-kit [1] [20] and to a lesser extent PDGFR [20]. Treating tumour xenografts with cediranib inhibits tumour growth by preventing angiogenesis and inducing a regression of immature vasculature within the tumour [1] [21], therefore cediranib has little effect in cell culture. As a result of the changes in the vasculature, genes associated with vessels would be predicted to be reduced, and genes associated with cellular stress associated feedback loops induced.

Using the two Calu-6 human lung tumour xenografts from animals treated with cediranib, both tumour and host gene expression changes between control and treated samples were measured using DESeq [22]. For the purpose of the functional analysis, significantly differentially expressed genes were defined as those achieving fold change magnitude>2 and *p*-value<0.1, resulting in 271 up-regulated and 771 down-regulated tumour genes in response to cediranib, and 192 up-regulated and 861 down-regulated host genes. Read counts across each human and mouse transcript for the two treated and two control samples are given in Tables S10 and S11 respectively. Fold changes and significance for the same transcripts are given in Tables S12 (human) and S13 (mouse). Included in these lists are a number of non-protein coding transcripts such as lncRNAs potentially involved in the response to cediranib and these are also indicated in Tables S12 and S13. Of the 271 up-regulated tumour genes, only 10 overlapped with a corresponding up-regulated host gene. Similarly only 52 down-regulated tumour genes overlapped with a corresponding down-regulated host gene, further highlighting the ability of the RNA-Seq approach to differentiate species-specific gene changes.

A survey of the list reveals a reduction in a number of host transcripts associated with endothelial cells or vasculature, consistent with the mode of action of cediranib. In addition to detecting a reduction in well described genes such as *Vegfr-2* (*Kdr;* log_2_ fold change = −1.21, *p* = 0.008) and *Dll4* (log_2_ fold change = −2.84, *p* = 0.03), a reduction in less well studied genes such as the *Clec14a* (log_2_ fold change = −1.77, *p* = 0.01), a tumour specific endothelial gene that regulates pro-angiogenic phenotypes [23], was also detected. Reduction in endothelial genes was associated with a concomitant up-regulation of murine genes associated with inflammation, stress response, and cell migration. An inflammatory response induced by inhibition of VEGF-signalling, and the subsequent recruitment of bone marrow derived cells such as CD11b+ Gr-1+ myeloid cells to the tumour microenvironment has been described as a resistance mechanism for VEGF-signaling inhibitors [24]–[26]. Although this is thought to be mediated by *Bv8* and *Gcsf*, a number of other inflammation genes such as *Ptgs2* (log_2_ fold change = 1.54, *p* = 0.0001) and *Il1b* (log_2_ fold change = 1.23, *p* = 0.04) were up-regulated, which may also function to recruit inflammatory cells or bone marrow derived cells to the tumour following treatment. The RNA-Seq analysis also revealed novel murine genes induced following cediranib treatment. One notable gene induced on treatment is *S100a9* recently shown to drive inflammatory cell mediated chemo-resistance in mouse syngeneic models [27].

Functional enrichment amongst genes showing significantly differential expression on cediranib treatment was assessed using IPA (Ingenuity® Systems, www.ingenuity.com). A threshold-free approach was also taken to assess functional enrichment using Gene Set Enrichment Analysis (GSEA, http://www.broad.mit.edu/gsea/index.jsp) [28], with genes ordered by –log_10_(*p*-value)×N where N = 1 for genes up-regulated in response to cediranib, and N = −1 for down-regulated genes.

**Figure 4:**
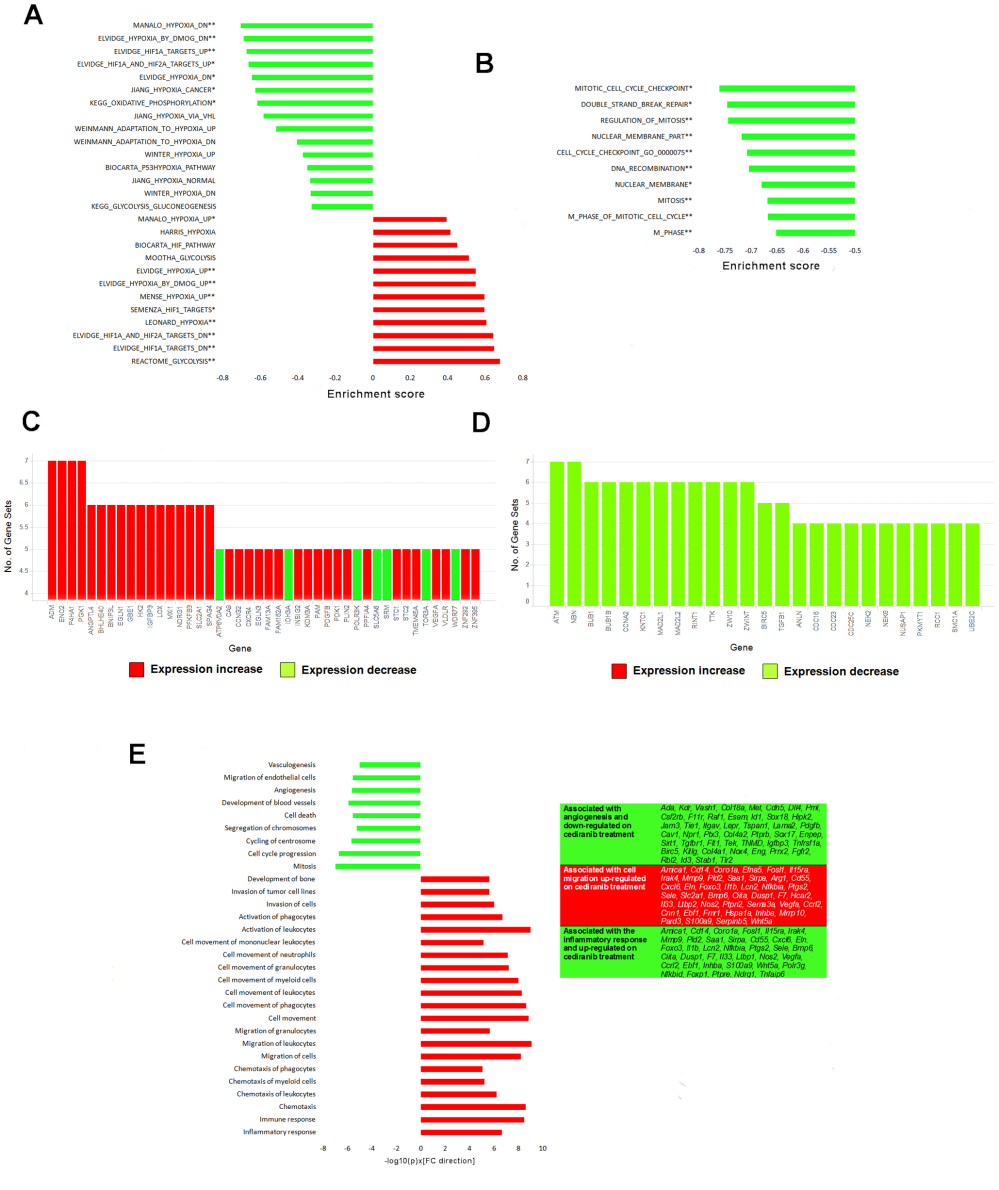
RNA-Seq differentiates the tumour (human) transcriptional response to cediranib from the host (mouse). Gene Set Enrichment Analysis (GSEA) reveals significant enrichment of (A) hypoxia and (B) cell cycle associated signatures in tumour genes differentially regulated in response to cediranib dosed at 6 mg/kg once daily for 4 days (** indicates gene sets enriched with *p*<0.001, FDR *q*<0.05 and FWER *p*<0.1; * indicates gene sets enriched with *p*<0.001 and FDR *q*<0.05). GSEA-defined “Leading Edge” genes most frequently included in (C) hypoxia-associated gene sets and up-regulated in response to cediranib include CA9, HK2 and VEGFA. Leading edge cell cycle associated genes are given in (D). (E) Ingenuity Pathway Analysis (IPA) highlights functions significantly enriched (*p*<1×10^−5^) amongst host genes differentially regulated in response to cediranib. For both GSEA and IPA, genes achieving a log_2_ fold change magnitude>1 and *p*<0.1 were defined as differentially regulated. *n* = 2 in treated and control groups; a positive fold change indicates genes up-regulated in response to cediranib, and *vice versa*.

GSEA revealed significant enrichment (GSEA *p*<0.001) of several hypoxia associated signatures in human tumour genes differentially regulated in response to cediranib (Figure 4A). Specifically, tumour genes up-regulated in response to cediranib were enriched with genes up-regulated in hypoxia, and vice versa (Figure 4B), including increased expression of glycolysis pathway genes and decreased expression of genes involved in oxidative phosphorylation. IPA analyses supported these findings, indicating an up-regulation of genes under the control of the transcription factors EPAS1 (IPA *p* = 4.19×10^−4^) and HIF1A (IPA *p* = 6.74×10^−4^), both of which are induced upon hypoxia, with a simultaneous down-regulation of genes involved in nitric oxide and reactive oxygen species production (IPA *p* = 2.06×10^−4^). Tumour genes down-regulated following cediranib treatment were strongly enriched for genes defined by the Gene Ontology [29] to be involved in the cell cycle consistent with the reduction in tumour growth (IPA *p* = 2.54×10^−5^; Figures 4C and 4D). In addition potential targets of the E2F transcription factor family were also modulated (IPA *p* = 4.14×10^−4^) consistent with an anti-proliferative effect in the tumour.

**Figure 5:**
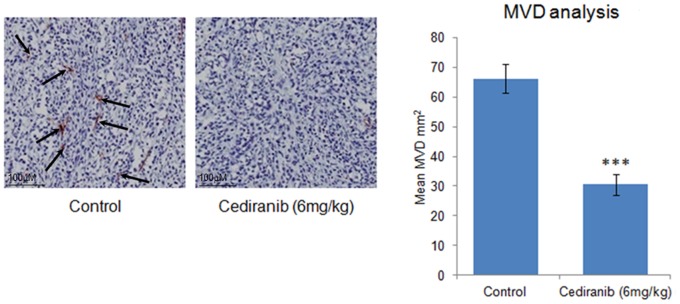
The phenotypic effects of cediranib observed by immunohistochemical analysis of supporting vasculature in cediranib treated tumours. Calu-6 xenografts were established and dosed for 4 days with cediranib at 6mg/kg once daily and fixed in formalin. Micro Vessel Density was then quantified by histological staining for CD31 as previously described [32]. Images are representative for CD31 staining in control and cediranib treated tumours, and arrows indicate blood vessels. Graph depicts the significant (*p*-value<0.001; student t-test) reduction of the supporting vasculature in cediranib treated tumours; 8 control tumours and 6 cediranib tumours, error bars are standard error of the mean.

IPA analysis revealed an up-regulation of genes involved in the inflammatory response (IPA *p* = 1.15×10^−9^), in particular leukocyte and phagocyte activation/migration (Figure 4E), supporting the finding by GSEA that genes associated with cytokine activity are increased following cediranib treatment (GSEA *p*<0.001). Up-regulation was also observed from genes associated with the transcription factor NFKB (IPA *p* = 3.57×10^−7^). Down-regulation was observed for host genes involved in blood vessel development (IPA *p* = 1.20×10^−6^), angiogenesis (IPA *p* = 2.32×10^−6^), vasculogenesis (IPA *p* = 9.67×10^−6^) and migration of endothelial cells (IPA *p* = 2.69×10^−6^; Figure 4E) coupled with reduced expression of genes involved in collagen connective tissue formation (GSEA *p*<0.001). GSEA also revealed a related to the down-regulation of endothelial genes. As in the human cells, the mouse compartment also showed down-regulation of genes involved in the cell cycle (IPA *p* = 1.02×10^−7^) and DNA repair pathways (IPA *p* = 5.41×10^−6^) following cediranib treatment. Thus, differentially expressed host genes showed clear enrichment for functions associated with the roles of VEGF in the cell. Immunohistochemical analysis confirmed the reduction of the supporting vasculature in cediranib treated tumours (Figure 5), which suggests that the broad pathway changes identified by RNA-Seq are translated into phenotypic effects.

## Discussion

Using current transcript based approaches to differentiate murine from human transcripts in human tumour xenografts and thus investigate the effects of therapeutic agents, or identify emerging resistance mechanisms that potentially limit efficacy has proven challenging. Standard microarray approaches are limited by their susceptibility to cross-species contamination, low dynamic range and biased transcript coverage. PCR based approaches offer an increase in dynamic range, but are limited to a fraction of the transcriptome even with new high-throughput techniques. In this study, we have demonstrated the application of RNA-Seq to xenograft profiling and its potential in differentiating the tumour and host components on a single platform. We show that RNA-Seq is highly comparable to a gold standard approach such as RT-qPCR in measuring species-specific transcriptional profiles, and that a simple adaptation to the standard mapping pipeline is sufficient to accurately quantify tumour and host specific gene expression. Whilst bias towards highly expressed mouse genes in our selection of transcripts for RT-qPCR profiling may have contributed to the high correspondence between the two platforms, our results strongly suggest that the approach will accurately measure the expression levels of low abundance transcripts given sufficient sequencing depth. A major technical challenge of profiling the mouse component of a tumour xenograft is the often high tumour content of the sample where a 10∶1 ratio of human to mouse RNA is typical. Therefore, we were encouraged by the high level of correspondence between RNA-Seq and RT-qPCR across mouse genes despite low sequencing depth and a significant percentage of potential mouse sequences lost as a result of cross-mapping to the human genome. Furthermore, meaningful separation of tumour and host gene changes upon cediranib treatment was achieved and RNA-Seq suggested a stromal response to the VEGF signaling inhibitor. However, since the mouse component typically consists of a mixed cell population, it is challenging to attribute mouse gene expression to stromal cells specifically. Higher coverage and longer read lengths are likely to yield more accurate results, greater species specificity and increase the possibility of detected splice variants, mutations and gene fusions in the host tissue, in addition to novel non-coding mouse RNA expression changes upon treatment However, despite rapid technological developments and decreasing costs of sequencing, low mouse coverage is likely to remain an issue for some time therefore our finding that valid information from the host can be sought even below recommended levels of sequencing depth is significant. Cost implications also continue to hinder the use of large numbers of replicates in RNA-Seq so we were further encouraged that using only two treated and two control animals generated relevant hypotheses. However, in general, we recommend that hypotheses generated from such low replicate numbers are subsequently validated with lower cost technologies and additional replicates to increase the likelihood of capturing the full extent of technical and biological variability.

Recently, a statistical method to pre-assign short reads to their species of origin prior to mapping has been proposed [30]. The study reports that whilst the method yields an improvement over standard mapping tool assignment, the proportion of reads misclassified by the latter is small, supporting our conclusion that by simply using the read classification implied from a standard mapping tool such as Bowtie [31], RNA-Seq can accurately differentiate tumour and host transcription. Our study also goes further by being the first to demonstrate that potentially relevant biology may be extracted from the mouse component, even at low sequencing depth, exemplified by accurate differentiation of the tumour/host response to cediranib.

The level of correspondence between RNA-Seq and the 3′ mRNA arrays representing both species suggests that the platforms show comparable performance in quantifying expression across both human and mouse transcripts. However, correspondence between RNA-Seq and the mouse array increased significantly when probes at risk of cross-hybridization were ignored suggesting that cross-species contamination is an issue on the gene array particularly across genes from the species representing the smaller RNA component. Nevertheless, arrays remain viable tools for genomic studies including the analysis of samples derived from xenografted tissues, particularly with the development of platforms such as the Illumina BeadChip Expression Array designed specifically to minimize cross-species hybridization [6] [15].

Whilst findings should be validated using larger sample numbers and lower throughput technologies such as PCR or Western Blotting, we believe the combination of RNA-Seq profiling and pathway mapping can generate rational hypotheses from both the tumour and host into the effect of treating tumours with drugs. Using cediranib as an example, reduction in a number of host transcripts associated with endothelial cells or vasculature was observed, consistent with the mode of action of cediranib. In additional, RNA-Seq revealed a number of novel host genes such as *Clec14a* and *S100a9*, as well non-protein coding transcripts such as lncRNAs potentially involved in the cediranib response. Further exploration of the host genes modified by cediranib will give broader insight into the types of vessels targetted, and more importantly the feedback loops induced following treatment. In conclusion, this study has shown that RNA-Seq is an effective strategy to generate hypotheses into both the mode of action, highlighting potential biomarkers of drug target inhibition, as well as induced responses following drug treatment.

## Materials and Methods

### Ethics Statement

Tumour xenograft tissue was derived from experiments conducted with strict adherence to licenses issued under the UK Animals (Scientific Procedures) Act 1986 and after local ethical review and approval. The study was performed under Home Office project license (PPL) number 40/3483.

### Tumour Xenograft Establishment and Immunohistochemistry

Cells for tumour implantation were grown in DMEM supplemented with glutamine and 10% foetal calf serum and implanted subcutaneously in nude mice as previously described [1]. Calu-6 xenografts were established in fourteen mice and eight animals dosed for 4 days with cediranib at 6 mg/kg once daily orally once tumours reached 0.2 cm^3^, and six animals with 1% polysorbate 80 vehicle alone as previously described [1]. Two control LoVo xenografts were also established subcutaneously and animals dosed with 1% polysorbate 80 vehicle. All control tumours were grown to between 0.8 and 1 cm^3^ then rapidly excised and snap frozen in liquid nitrogen. CD31 immunohistochemical staining and microvessel density analysis on the fourteen Calu-6 xenografts was performed as described in [32]. Two treated xenografts with the lowest microvessel density and two control xenografts with the highest microvessel density were selected for RNA-Seq with the purpose of method testing and evaluation.

### RNA Extraction

∼50 mg of tissue were cut from the frozen tumours and RNA isolated using the RNeasy Lipid Tissue Mini Kit (Qiagen) according to manufacturer’s instructions. On-column DNase digestion was performed using the RNase-free DNase Kit (Qiagen). RNA concentration was measured using the NanoDrop ND1000 (NanoDrop), and quality determined using the Agilent RNA nano 6000 kit and Bioanalyzer (Agilent Technologies). RNA integrity numbers (RIN) for all samples fell between 7 and 10.

### Human-specific and Mouse-specific TaqMan® Fluidigm Profiling

Human and mouse specific TaqMan Gene Expression Assays were designed and supplied by Applied Biosystems, and eukaryotic 18S rRNA was used as the endogenous control. Each of the assays was validated for species specificity by cross screening versus both a human and mouse mRNA reference library containing the combined mRNA context of 10 human and mouse cell lines [19]. Reverse transcription was performed with 50 ng of total RNA in a final volume of 20 µl, using the High Capacity cDNA Reverse Transcription kit (Applied Biosystems). The following thermal profile was used: 25°C for 10 minutes, 37°C for 120 minutes, 85°C for 5 seconds and 4°C for 2 minutes. Pre-amplification was performed with 1.25 µl of resulting cDNA in a final volume of 5 µl, using a pool of TaqMan assays at a final dilution of 1 in 100 and TaqMan PreAmp Master Mix (Applied Biosystems). The following thermal profile was used: 95°C for 10 minutes, 14 cycles of 95°C for 15 seconds and 60°C for 4 minutes. Pre-amplified samples were diluted 1 in 5 with TE prior to performing qPCR on the Fluidigm BioMark System.

Sample and assay preparation for 48.48 Fluidigm Dynamic Arrays were performed according to manufacturer’s instructions. Briefly, samples were mixed with DA Sample Loading Reagent (Fluidigm), and TaqMan Gene Expression Master Mix (Applied Biosystems). Assays were mixed with DA Assay Loading Reagent (Fluidigm). The 48.48 Fluidigm Dynamic Arrays were primed and loaded on an IFC Controller (Fluidigm) and qPCR was performed on a BioMark System (Fluidigm) using the following thermal profile: 50°C for 2 minutes, 95°C for 10 minutes and 40 cycles of 95°C for 15 seconds and 60°C for 1 minute. Data were collected and analysed using the Fluidigm BioMark Real-Time PCR Analysis software.

Species-specific normalisation of the expression data to 18S rRNA was achieved by correcting the eukaryotic 18S rRNA Ct values measured using TaqMan assay Hs99999901_s1 (Applied Biosytems) for the proportion of human and mouse 18S rRNA in each xenograft sample [19]. Gene expression values were calculated using the comparative CT (−ΔCT) method as previously described in User Bulletin #2 ABI PRISM 7700 Sequence Detection System 10/2001, using the corrected 18S rRNA Ct values for normalisation. Genes altered by cediranib treatment were determined by student t-tests.

### Experimental Estimation of Species Content of Sample

The proportions of human and mouse 18S rRNA were determined using species-specific ARMS™ TaqMan RT-qPCR assays designed in-house. Human and mouse 18S rRNA ARMS assay primer and probe sequences are given in Table S14. Reverse transcription was performed with 1 ng total RNA in a final volume of 50 µl using the in-house 18S rRNA reverse primer which reacts with both human and mouse sequence at a final concentration of 0.5 µM and the TaqMan Reverse Transcription Kit (Applied Biosystems). The reaction was performed on the ABI PRISM 7700 Sequence Detection System (Applied Biosystems) using the following thermal profile: 25°C for 10 minutes, 48°C for 30 minutes, 95°C for 5 minutes and 4°C for 2 minutes. qPCR was performed with 5 µl resulting cDNA in a final volume of 25 µl using the in-house, species-specific 18S rRNA ARMS assays at a final concentration of 0.2 µM each primer and 0.1 µM probe and TaqMan Universal PCR Master Mix (Applied Biosystems). The PCR was performed on the ABI PRISM 7700 Sequence Detection System (Applied Biosystems) using the following thermal profile: 50°C for 10 minutes, 94°C for 20 minutes, and 40 cycles of 95°C for 20 seconds and 60°C for 1 minute.

Human and Mouse Genomic DNA (Promega) and Human and Mouse Universal Reference RNA (Stratagene) were used to confirm assay specificity. Standard curves, generated using Human and Mouse QPCR Reference Total RNA (Stratagene), were used to determine the proportions of human and mouse 18S rRNA in each xenograft sample. This method was validated by determining the relative amounts of human and mouse 18S rRNA in known synthetic mixtures of human and mouse Total RNA. Predictions correlated closely with the known values (r^2^ = 0.99). The Ct value obtained for each xenograft sample from the eukaryotic 18S rRNA assay Hs99999901_s1 was corrected for the proportion of human and mouse 18S rRNA present by dividing this Ct value by the proportion on the linear scale.

### RNA Library Preparation for Sequencing

Total RNA was further cleaned using the Ribominus concentration module (Invitrogen) according to manufacturer’s instructions. rRNA depletion was carried out with the Dynabeads mRNA purification kit (Invitrogen) according to manufacturer’s instructions. Successful removal of rRNA was confirmed using the Bioanalyzer. Libraries enriched with polyA mRNA suitable for sequencing by the SOLiD platform were created using the SOLiD Total RNA Seq Kit supplied by Applied Biosystems in combination with the SOLiD RNA Barcoding Kit, Module 1–16. In each instance, RNase III digested polyA RNA was used as input into library creation and 15 cycles of amplification were employed to produce the final libraries. These libraries were sequenced on the SOLiD platform (Applied Biosystems). Initially, sequencing of the 10 bp library barcode was performed followed by 35 bp reverse and 50 bp forward paired-end sequencing. Only the 50 bp forward reads were used in this study.

### Microarray Preparation and Data Analysis

100 ng of RNA was hybridised on both the Affymetrix GeneChip U133 Plus 2 and Mouse Genome 430 2.0 GeneChips® with 3′ IVT Express Labeling Kits. All microarray procedures were performed at AROS, Denmark following manufacturer instructions. Robust Multi-array Average (RMA) normalization on the raw CEL files was carried out using the R Bioconductor package “affy” [33]. Probeset to gene level mappings and gene expression summarization were performed using the R Bioconductor package “xmapcore” [34].

In order to identify the Human U133 Plus 2.0 probesets most susceptible to cross-hybridisation from mouse mRNA, we performed similarity searches between all oligonucleotide 25-mer probe sequences and the mouse transcriptome as defined by Ensembl version 63 using Bowtie [31] in *-v* alignment mode with a tolerance of 3 mismatches. The whole procedure was then repeated for Mouse 430 2.0 array probesets against the human transcriptome. All genes mapped by probesets containing probe “hits” were defined as susceptible to cross-species signals. 92,108/496,468 mouse array probes (18.6%) mapped to the human transcriptome with at least 3 mismatches corresponding to 21,953/45,101 (48.7%) probesets and 13,245/18,109 (73.1%) genes. 109,075/604,258 human array probes mapped to the mouse transcriptome corresponding to 24,448/54,675 (44.7%) probesets and 14,282/20,808 (68.6%) genes.

### Species-specific Genome Alignment and Annotation

Reads of length 50 bases were aligned to the human (GRCh37/hg19) and mouse (NCBI37/mm9) genomes separately using Bowtie [31] in *-n* alignment mode. In both cases, reads were also mapped to a species-specific exon junction database simultaneously with the genome (see below). Seed length was set to 25 bases with no more than 2 mismatches allowed in this region. The first and last bases were ignored as they are supported by only one colour, and five bases were trimmed from the low quality 3′ end of each read giving an effective read length of 43 bases. The “tryhard” parameter was set to “true”, and only uniquely mapped reads were considered so the *-m* option was set to 1. All other parameters were set to their defaults. A custom Perl script was used to remove reads that mapped to both human and mouse genomes. To avoid ambiguous assignment of intron-spanning reads to pseudogenes [35], we mapped reads simultaneously across the genome and a species-specific splice junction database, thus reads mapping to both a pseudogene and splice junction were classed as a non-uniquely mappable and discarded. Non-redundant datasets of 336,102 and 248,958 known human and mouse exon-exon junctions respectively were generated using exon coordinates downloaded from Ensembl version 63. To ensure that a 43 base read overlapped a splice junction, only the last 42 bases of the first exon and the first 42 bases of the second exon were considered (if the exon length exceeded 42 bases). An additional ∼6% (∼1.2 M reads) and ∼8% (∼150 K reads) of reads mapped in the sense orientation to our human and mouse splice junction datasets respectively (Table S15). Of these hits, only ∼0.01% (∼150 reads) and 0.04% (∼70 reads) mapped in the antisense direction and were discarded as they were considered false positive hits. Raw sequence data are available in the ArrayExpress database (www.ebi.ac.uk/arrayexpress) under accession number E-MTAB-1627.

### Measurement of Expression Level

Bowtie alignment format was converted to BED format using a custom Perl script, and the intersectBed tool from the BEDtools suite of software was used to overlap the mapped reads with.gtf annotation files based on Ensembl version 63. From the overlap files, the number of reads mapping to each Ensembl transcript was calculated. We adapted the RPKM measure of [36] to calculate a normalized expression level based on the read count across the transcript:
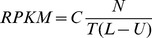
(1)where *N* is the number of reads mapping to the region, *L* is the region length, *U* is the number of non-unique loci across the transcript (see below), *T* corresponds to the total number of uniquely mappable reads in each cell line, and *C* is a constant set to 1×10^6^ in this study. To avoid taking logs of zero, we added a small constant (0.001) to the RPKM.

### Non-unique Loci Identification

There are a significant number of positions where, for a given 50-base sequence, one or more identical 50-mers are found elsewhere on the genome. At these loci, the probability of finding a uniquely-mapped read given our mismatch criteria is lower than at other positions, possibly preventing the detection of expression at these sites. Whilst attempts have been made to record the “uniqueome” of individual species [37], a combined human-mouse uniqueome applicable to xenograft RNA-Seq is lacking. Therefore, we generated our own in-house dataset by performing an exhaustive all-against-all search for all non-unique 50-mers across a concatenated human-mouse genome and recorded where they matched against the reference. In detail, starting from each base position on a chromosome we took a 50 base region of consecutive sequence and searched for an identical 50 base match elsewhere on the concatenated genome. If one or more matches were found then that base position and all start positions of the matches were marked with a “1”, otherwise the base position was marked with “0”. In this way, a profile of “ones” (corresponding to non-unique loci) and zeros (unique-loci) was a generated for each chromosome in human and mouse. 15% and 18% of human and mouse genomic loci respectively were defined as non-unique using these criteria.

### Differential Expression in RNA-Seq

We used the R Bioconductor package DESeq [22] to identify genes differentially expressed between cediranib treated and control samples. DESeq uses a model based on the negative binomial distribution, a generalisation of the Poisson model that allows modelling of biological as well as technical variance. Count data were uploaded into the R session in the form of a tab-delimited table with five columns with the first column indicating gene identity, and the others representing reads counts in Control_1, Control_2, VEGFi_1 and VEGFi_2. Size factors (effective library sizes) were calculated by dividing the library size of each sample by the median library size of all four samples. All other DESeq package functions were applied using default parameters.

### Correspondence Score

To measure detection correspondence between RNA-Seq and RT-qPCR we used a modified version of the Matthew’s Correlation Coefficient to calculate a correspondence score, CS [38]–[39]

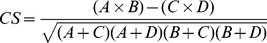
(2)where A indicates the number of genes detected on both platforms, B indicates the number of genes undetected on both platforms, C indicates the number of genes detected by RNA-Seq but undetected by RT-qPCR, and D indicates the number of genes undetected by RNA-Seq but detected by RT-qPCR. A CS of -1 means that all genes detected by RNA-Seq are undetected by RT-qPCR and vice versa, a CS of zero means that correspondence is no better than random, and a CS of 1 indicates perfect correspondence.

## Supporting Information

Figure S1 **Strong correlation between RNA-Seq versus experimentally determined species content of RNA sample.** For RNA-Seq, percentages are based on the proportion of reads mapping uniquely to mouse. Experimental procedure for confirming species content is given in Materials and Methods.
(TIFF)

Figure S2 **RNA-Seq gene expression correspondence between biological replicates.** Scatterplots (A) and (B) represent tumour (human) gene expression from control (*r^2^* = 0.93) and cediranib treated (once daily orally at 6 mg/kg; *r^2^* = 0.90) animals respectively. Scatterplots (C) and (D) represent host (mouse) gene expression in control (*r^2^* = 0.73) and cediranib treated (*r^2^* = 0.68) animals respectively. *r^2^* values are calculated from genes detected in both replicates. Gene expression is given as RPKM.
(TIFF)

Figure S3 **RT-qPCR gene expression correspondence between biological replicates.** Scatterplot (A) represents tumour (human) gene expression from control animals (*r^2^* = 0.95) and (B) corresponding host (mouse) gene expression (*r^2^* = 0.88). *r^2^* values are calculated from genes detected in both replicates. Gene expression is given as –ΔCT.
(TIFF)

Figure S4 **Changes in human and mouse gene detection correspondence between RNA-Seq and RT-qPCR at count thresholds ranging from one to 101 reads.** Correspondence at each threshold was calculated using the Correspondence Score described in Materials and Methods. Data for Human and Mouse Control_2 samples are shown.
(TIF)

Table S1 **Calu6 xenograft mapping coverage statistics.**
(XLSX)

Table S2 **Calu6 xenograft transcript mapping statistics.**
(XLSX)

Table S3 **Calu6 xenograft protein coding transcript coverage.**
(XLSX)

Table S4 **Correspondence between the proportion of reads mapping to mouse and the experimentally estimated species content of sample.**
(XLSX)

Table S5 **RT-qPCR assay probe identifiers and -ΔCT values across control and treated calu6 xenograft samples.**
(XLSX)

Table S6 **Correspondence in gene expression quantification and detection between RNA-Seq and RT-qPCR.**
(XLSX)

Table S7 **RT-qPCR ΔCT values for all six treated and eight control calu6 xenograft samples.**
(XLSX)

Table S8 **Correspondence in gene expression quantification between RNA-Seq and Affymetrix Human U133 Plus 2.0/Mouse 430 2.0 gene arrays.**
(XLSX)

Table S9 **LoVo xenograft mapping coverage statistics.**
(XLSX)

Table S10 **Read counts across human transcripts in control and treated calu6 xenograft samples.**
(XLSX)

Table S11 **Read counts across mouse transcripts in control and treated calu6 xenograft samples.**
(XLSX)

Table S12 **Fold changes and significance across human transcripts between control and treated calu6 xenograft samples.**
(XLSX)

Table S13 **Fold changes and significance across mouse transcripts between control and treated calu6 xenograft samples.**
(XLSX)

Table S14 **Human and mouse 18S rRNA ARMS assay primer and probe sequences.**
(XLSX)

Table S15 **Calu6 xenograft splice junction read statistic.**
(XLSX)
