# Supplementary figures and images for: RNA-Seq Differentiates Tumour and Host mRNA Expression Changes Induced by Treatment of Human Tumour Xenografts with the VEGFR Tyrosine Kinase Inhibitor Cediranib
Source: PLoS One. 2013 Jun 19;8(6):e66003. doi: 10.1371/journal.pone.0066003 (PMC3686868; doi:10.1371/journal.pone.0066003)

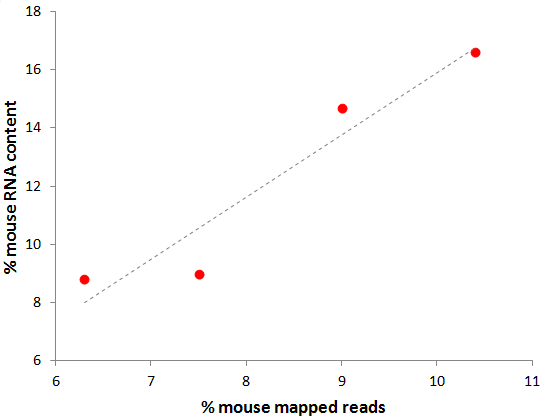

Supplement: Figure S1 — Strong correlation between RNA-Seq versus experimentally determined species content of RNA sample. For RNA-Seq, percentages are based on the proportion of reads mapping uniquely to mouse. Experimental procedure for confirming species content is given in Materials and Methods. (TIFF) [file pone.0066003.s001.tiff]

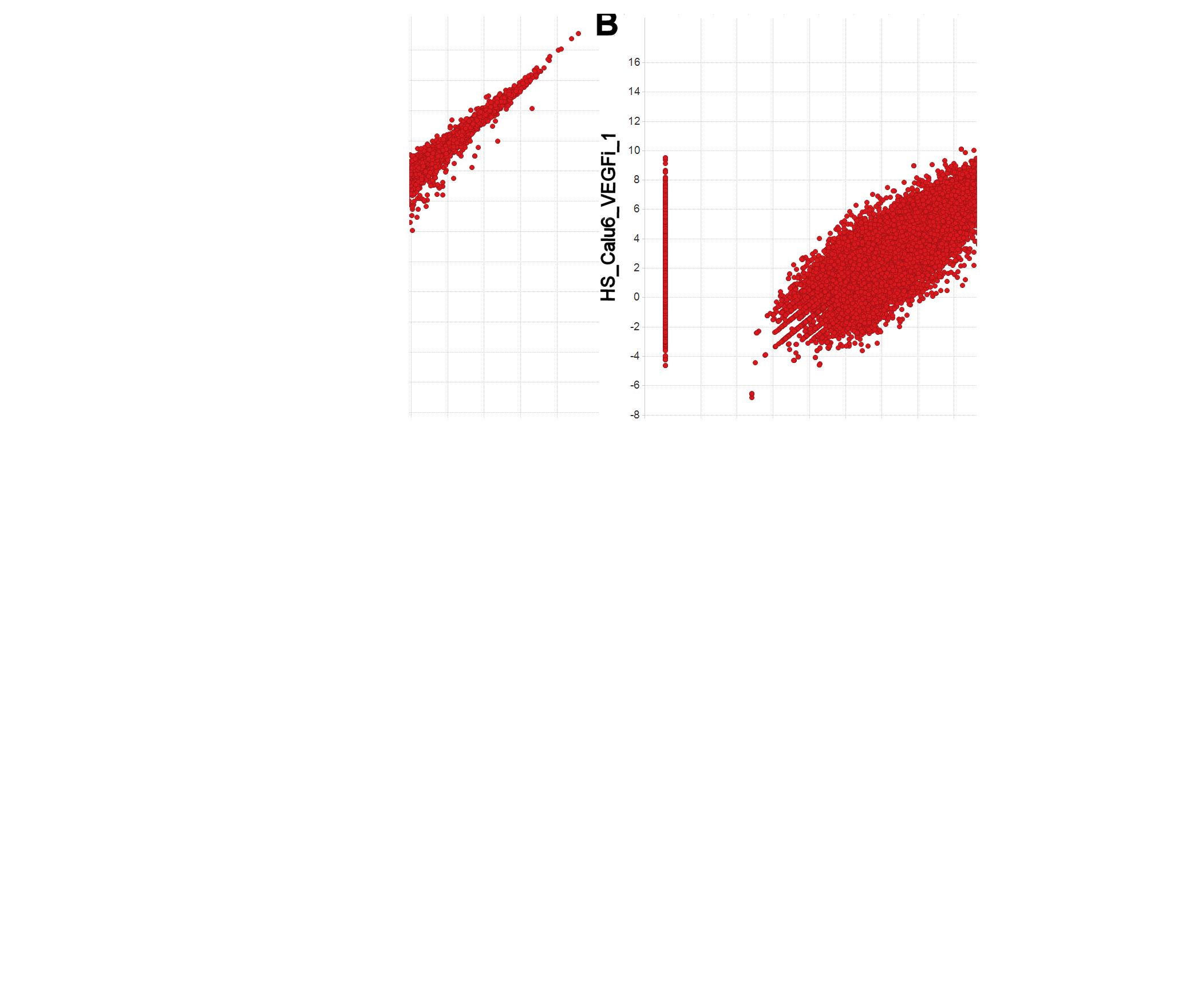

Supplement: Figure S2 — RNA-Seq gene expression correspondence between biological replicates. Scatterplots (A) and (B) represent tumour (human) gene expression from control (r2 = 0.93) and cediranib treated (once daily orally at 6 mg/kg; r2 = 0.90) animals respectively. Scatterplots (C) and (D) represent host (mouse) gene expression in control (r2 = 0.73) and cediranib treated (r2 = 0.68) animals respectively. r2 values are calculated from genes detected in both replicates. Gene expression is given as RPKM. (TIFF) [file pone.0066003.s002.tiff]

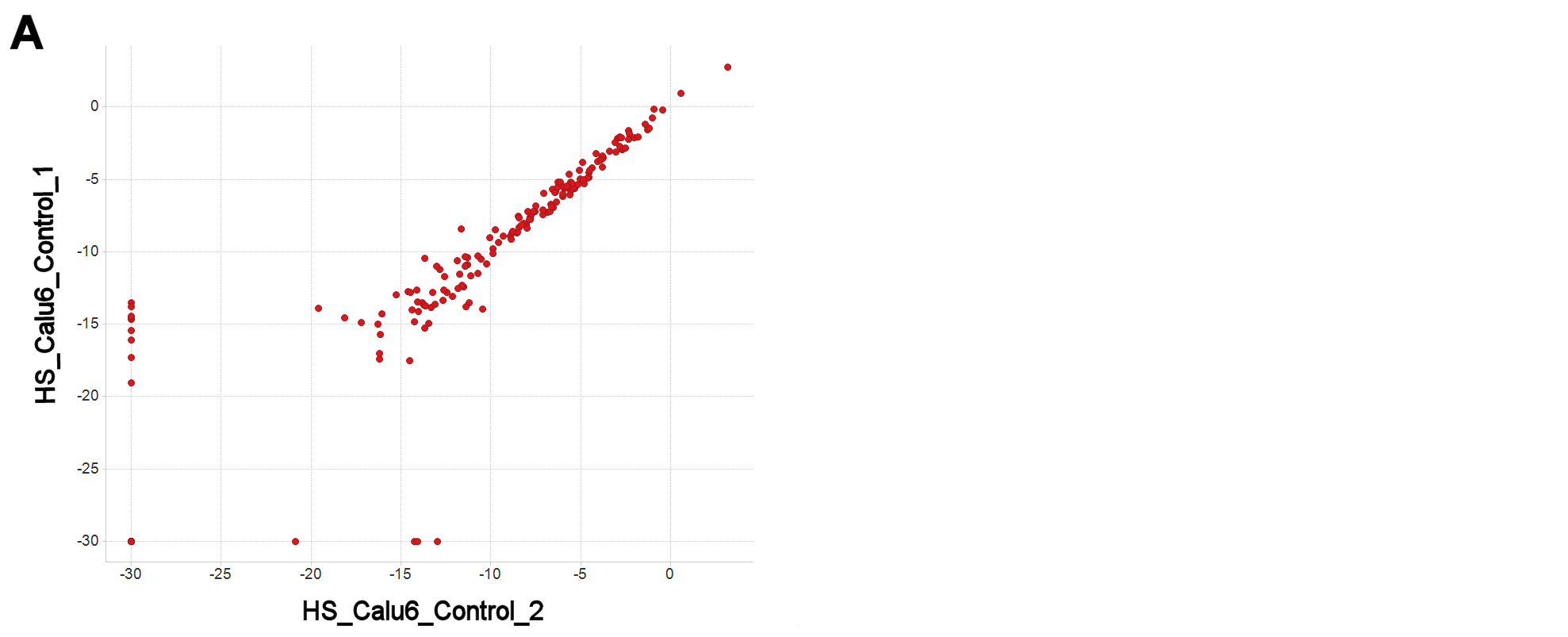

Supplement: Figure S3 — RT-qPCR gene expression correspondence between biological replicates. Scatterplot (A) represents tumour (human) gene expression from control animals (r2 = 0.95) and (B) corresponding host (mouse) gene expression (r2 = 0.88). r2 values are calculated from genes detected in both replicates. Gene expression is given as –ΔCT. (TIFF) [file pone.0066003.s003.tiff]

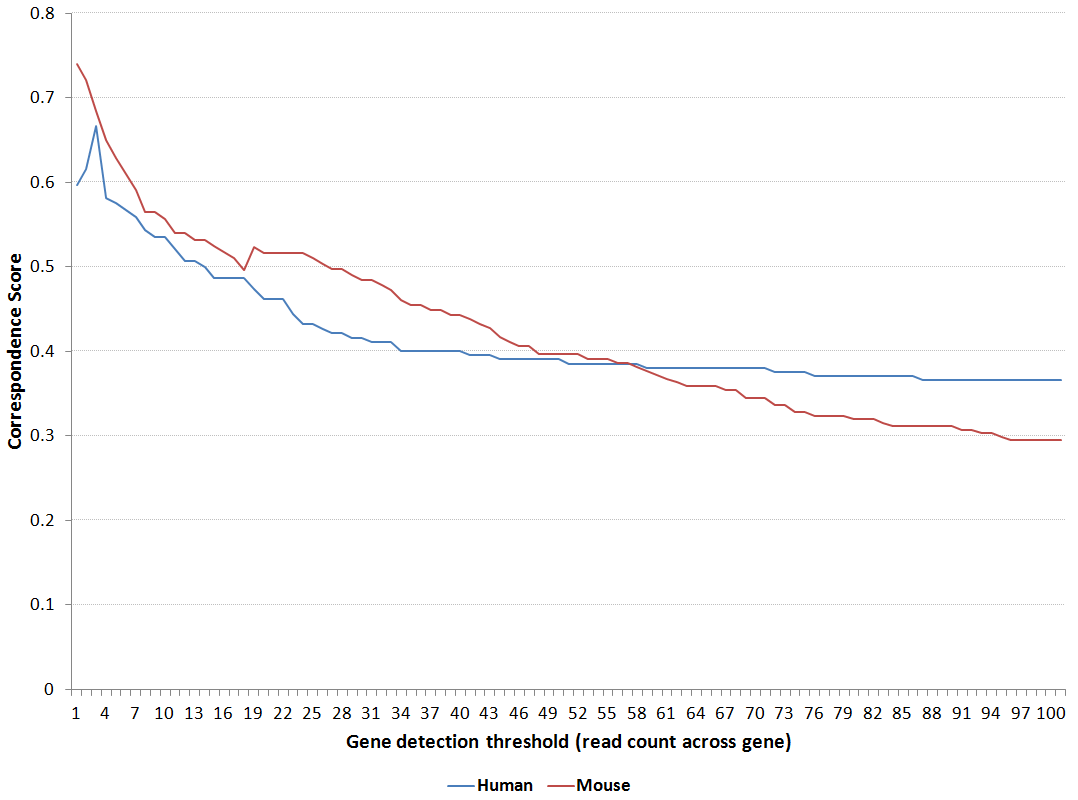

Supplement: Figure S4 — Changes in human and mouse gene detection correspondence between RNA-Seq and RT-qPCR at count thresholds ranging from one to 101 reads. Correspondence at each threshold was calculated using the Correspondence Score described in Materials and Methods. Data for Human and Mouse Control_2 samples are shown. (TIF) [file pone.0066003.s004.tif]
